# Polypharmacology-based approach for screening TCM against coinfection of *Mycoplasma gallisepticum* and *Escherichia coli*

**DOI:** 10.3389/fvets.2022.972245

**Published:** 2022-09-26

**Authors:** Jiaxin Bao, Yuan Wang, Shun Wang, Dong Niu, Ze Wang, Rui Li, Yadan Zheng, Muhammad Ishfaq, Zhiyong Wu, Jichang Li

**Affiliations:** ^1^College of Veterinary Medicine, Northeast Agricultural University, Harbin, China; ^2^College of Computer Science, Huanggang Normal University, Huanggang, China; ^3^Institute of Chinese Materia Medica, Heilongjiang Academy of Chinese Medicine Sciences, Harbin, China; ^4^Heilongjiang Key Laboratory for Animal Disease Control and Pharmaceutical Development, Harbin, China

**Keywords:** polypharmacology, Traditional Chinese Medicine, transcriptomics, multiple targets, network pharmacology

## Abstract

Natural products and their unique polypharmacology offer significant advantages for finding novel therapeutics particularly for the treatment of complex diseases. Meanwhile, Traditional Chinese Medicine exerts overall clinical benefits through a multi-component and multi-target approach. In this study, we used the previously established co-infection model of *Mycoplasma gallisepticum* and *Escherichia coli* as a representative of complex diseases. A new combination consisting of 6 herbs were obtained by using network pharmacology combined with transcriptomic analysis to reverse screen TCMs from the Chinese medicine database, containing *Isatdis Radix, Forsythia Fructus, Ginkgo Folium, Mori Cortex, Licorice*, and *Radix Salviae*. The results of therapeutic trials showed that the Chinese herbal compounds screened by the target network played a good therapeutic effect in the case of co-infection. In summary, these data suggested a new method to validate target combinations of natural products that can be used to optimize their multiple structure-activity relationships to obtain drug-like natural product derivatives.

## Introduction

Polypharmacology has been widely recognized as a new approach of modern drug discovery (1, 2), which might enable natural products to surpass the use of the traditional single-target drugs in terms of efficiency (3). Natural products with polypharmacological profiles show a promising prospect for the development of novel therapeutics for various complex diseases (4–6). The methods commonly used in polypharmacology include Molecular docking (7, 8), Network-based approaches (9, 10), and Omics-based systems biology approaches (11–13) along with the development of *in silico* pharmacology (14). In comparison to modern medicine, one of the advantages of TCM is the principle of herb compatibility, known as TCM formulae. However, the rationale of combining specific herb combinations remains unclear, and the space of herb combinations can be explored more effectively by using network pharmacology method (15, 16). More and more research methods on Chinese herbal compounds have been reported in recent years. Pan et al.'s study focused on the intervention of Huanglian Decoction in rats with type 2 diabetes mellitus by the methods of network pharmacology and metabolomics (17). Zhou et al. probed into the effect of Liuwei Dihuang decoction on the neuroendocrine immunomodulation network (18). In the present study, network pharmacology and systems biology will enable us to deliberately design lead molecules with expected polypharmacology as well as offer opportunities for TCM formula repurposing.

Due to the intensification of commercial poultry production, explosive multiple respiratory infections have become an urgent problem (19, 20). This co-pathogenesis is characterized by complex interactions between co-infection pathogens and the host (21, 22). Combined with the previous researches on the co-infection of *Mycoplasma gallisepticum* (MG) and *Escherichia coli* (*E. coli*) in our laboratory (20, 23), we tried to screen a new Chinese medicinal compound (NCMC) with the method of polypharmacology. There are many examples of natural products being used in drug discovery efforts that are directed at a wide range of indications beyond their traditional strengths with the development of research technologies (24–27). The study could establish a new methodology for the research of Chinese herbal medicinal extract and provide a new multi-target treatment method for the occurrence of co-infection.

## Materials and methods

### Bacterial infection and experimental groupings

MG R_low_ (*Mycoplasma gallisepticum* strain R) strain was obtained from the Harbin Institute of Veterinary Medicine (Chinese Academy of Agricultural Sciences), which was grown in a Modified Hayflicks medium. *Escherichia coli* O78 was isolated from chickens infected with colibacillosis in our laboratory and cultured in Nutrient Broth (Beijing Aoboxing BIO-TECH Co., Ltd.). The concentration of MG and *E. coli* were 1 × 10^9^ CCU/ml and 10^9^ CFU/ml, respectively. The detection of the density for MG and *E. coli* were consistent as explained in our previous study (23, 28).

Forty (1 day old) commercial Leghorn chickens were obtained from Chia Chau Chicken Farm (Harbin, Heilongjiang, China) and were assigned randomly to 2 groups (5 replicates of 20 chickens per group) namely the Control group and Co-infection group as described in our earlier study (23), (A) Control group: Fed only basal diet; (B) Co-infection group: 0.2 ml of MG medium (1 × 10^9^ CCU/ml) was injected into the left caudal thoracic air sac on the 7th day, and 0.1 ml of *E. coli* bacteria (10^9^ CFU/ml) was injected intraperitoneally on day 10. On the 13th day, 20 chickens from each group were euthanized using the method of cardiac blood collection. Lung samples in each group were collected for RNA-seq, while the serum samples were collected for non-targeted metabolomics.

### Collection of co-infection target genes and construction of TCM-target network

RNA was extracted by Trizol reagent (Invitrogen Inc., Carlsbad, CA) from lung tissue and was utilized to construct the final library (BGISEQ-500 RNA-Seq Library) (23). DEG-seq (Differentially Expressed Gene-sequencing) method was based on the Poisson distribution (Fold Change > 2 and Adjusted *P*-value < 0.001) (29, 30). According to the KEGG (Kyoto Encyclopedia of Genes and Genomes) annotation results and the official classification, we separately classified the functional and biological pathways of the DEGs. The DEGs from different sub-categories were compared to the STRING database by DIAMOND (www.diamondsearch.org/) (31). To obtain PPI (Protein-protein Interaction) diagram, the gene interactions were obtained by homology with known proteins. The network relations with a score ≥ 300 were screened out for mapping. After that, the genes with the highest score in each PPI diagrams were selected as target genes and used for subsequent network construction.

In the process of network construction, we first collected all the information of the TCMSP database (http://tcmspw.com/tcmsp.php) and obtained the total network of “TCM-component-target”, which was submitted in Supplementary material 1. We then put the collected target genes information into the total network for the reverse screening of TCM. The TCM was sorted according to the correlation degree of nodes in the reverse screening network. Furthermore, we classified the TCM based on different functions (Chinese Pharmacopoeia 2015 Edition) and chose the top1 of each classification, including *Isatdis Radix, Forsythia Fructus, Ginkgo Folium, Mori Cortex*, Licorice, and *Radix Salviae*. The details for these single Chinese medicinal herbs and materials and their roles based on TCM theory are listed in Supplementary material 2. Finally, the TCM-component-target network was established using Cytoscape 3.6.1 software (Bethesda, MD, USA).

### Preparation of NCMC

To verify whether the selected compound has a better therapeutic effect and determine the proportion of six herbs. Hence, we had made a treatment experiment based on the uniform design with the methods of pharmacodynamics and minimum inhibitory concentration (MIC). The multi-nonlinear regression equation was established by data processing system according to the comprehensive index. The results showed that the ratio of *Isatdis Radix, Forsythia Fructus, Ginkgo Folium, Mori Cortex*, Licorice, and *Radix Salviae* were 14:7:11:12:5:3 showed effective treatment. The design optimization details of NCMC for the treatment of co-infection was provided in Supplementary material 3.

Six herbs were purchased from Runhe Chinese medicine processing plant Ltd. Aqueous extract of NCMC was prepared as the following procedure. The medicinal materials were mixed in proportion and were macerated for 1 h in 10-folds distilled water (v/w), and then decocted for 1 h, after which the filtrate was collected and the residue was decocted again for 1 h up to 6-folds (v/w) in distilled water. The filtrates were mixed and condensed and then dried by vacuum-drier at 60°C (32, 33). The final concentration of the aqueous extract is 1 mg/mL.

### UHPLC-QTOF-MS analysis for components quantification

100 μL sample was added to 400 μL of an extracted solution containing 1.25 μg/mL of internal standard which was dissolved in water. After 30 s vortex, the samples were sonicated for 10 min in an ice-water bath. After the samples were incubated at −40°C for 1 h, then the sample was centrifuged at 12,000 rpm for 15 min at 4°C. Finally, the supernatant was put in a fresh 2 mL tube and 200 μL was transferred to a fresh glass vial for LC-MS/MS analysis (34, 35). LC separation was performed on the Nexera UHPLC LC-30A system (SHIMADZU, Japan) with a Waters BEH C18 column (1.7 μm^*^2.1^*^100 mm, Waters, USA). Mobile phases, water with 0.1% formic acid (A) and acetonitrile (B), were applied with gradient elution and the flow rate was kept at 0.4 mL/min. AB 5600 Triple TOF system (SCIEX, USA) was used to collect primary and secondary MS data based on IDA function under the control software (Analyst TF 1.7 (AB Sciex). Instrument dependent parameters: curtain gas = 35 psi, IonSpray voltage = +5,500 (POS)/−4,000 (NEG) V, nebulizer gas = 55 psi, heater gas = 55 psi, source temperature = 550°C. The original mass spectrometry data was imported using Progenesis QI software. The corresponding TCM metabolic database in the compound was established, and the peaks containing MS/MS data were identified by the self-built secondary mass spectrometry database (Biotree Biomedical Technology Co., LTD, China).

### NCMC treatment and groupings

A total of 120 White Leghorn chickens was purchased from Chia Chau Chicken Farm (Harbin, Heilongjiang, China), which were divided randomly into 4 groups as follows. Control group (A): Chickens in the Control group were fed in the same environment and kept until the end of experiments; Co-infection group (B): The co-infection model was constructed as previously; Co-infection + NCMC administration group (C): The same infection model as mentioned, then treated with the aqueous extract of NCMC, and given orally by gavage. The treatment started on day 13 and continued for 5 days, once a day at a dose of 450 mg/kg; NCMC control group (D): The same dose of NCMC (450 mg/kg) was given orally to chickens by gavage started at day 13 and continued for 5 days. On the 18th day, 20 chickens from each group were euthanized using the method of cardiac blood collection. Lung, tracheal, and serum samples in each group were collected for further analysis.

### Gross and microscopic examination

The severity of the gross air sac lesions and tracheal lesion scores were calculated on the basis of 0–3 scoring system as mentioned in our previous study (36). The tracheal tissues were fixed in 10% formalin, dehydrated, and immersed in the transparent samples of wax, cut into slices (4 μm), stained with hematoxylin and eosin (H&E). Secondly, the tracheal tissues were trimmed into small pieces of 1 mm^3^ and fixed overnight in 2.5% glutaraldehyde. They were washed with PBS twice and post-fixed in 1% osmium tetroxide at 4°C for 1 h. Next, the tissues were dehydrated by ethanol series and 100% acetone, embedded in epoxy resins. The ultrathin sections were stained with uranyl acetate and lead citrate and then observed under a GEM-1200ES transmission electron microscope (JEOL Ltd., Tokyo, Japan). Lastly, 1 mm^3^ piece of tracheal tissues was also observed under scanning electron microscopy (SEM, SU8010, HITACHI Ltd., Japan).

### Statistical analysis

The KEGG classification and the PPI maps were made by the BGI data mining online website (http://report.bgi.com). NCMC component classification diagram was made by RAW graphs (https://rawgraphs.io/). The data of microscopic lesion scores were analyzed with the Mann–Whitney *U*-test and the other data were analyzed with the one-way analysis method of the GraphPad Prism (version 8.02), using Tukey's student test method. The value of *P* ≤ 0.05 is considered statistically significant and *P* ≤ 0.01 as extremely significant.

## Results

### Transcriptome sequencing of co-infection group and targets screening

**Figure 1:**
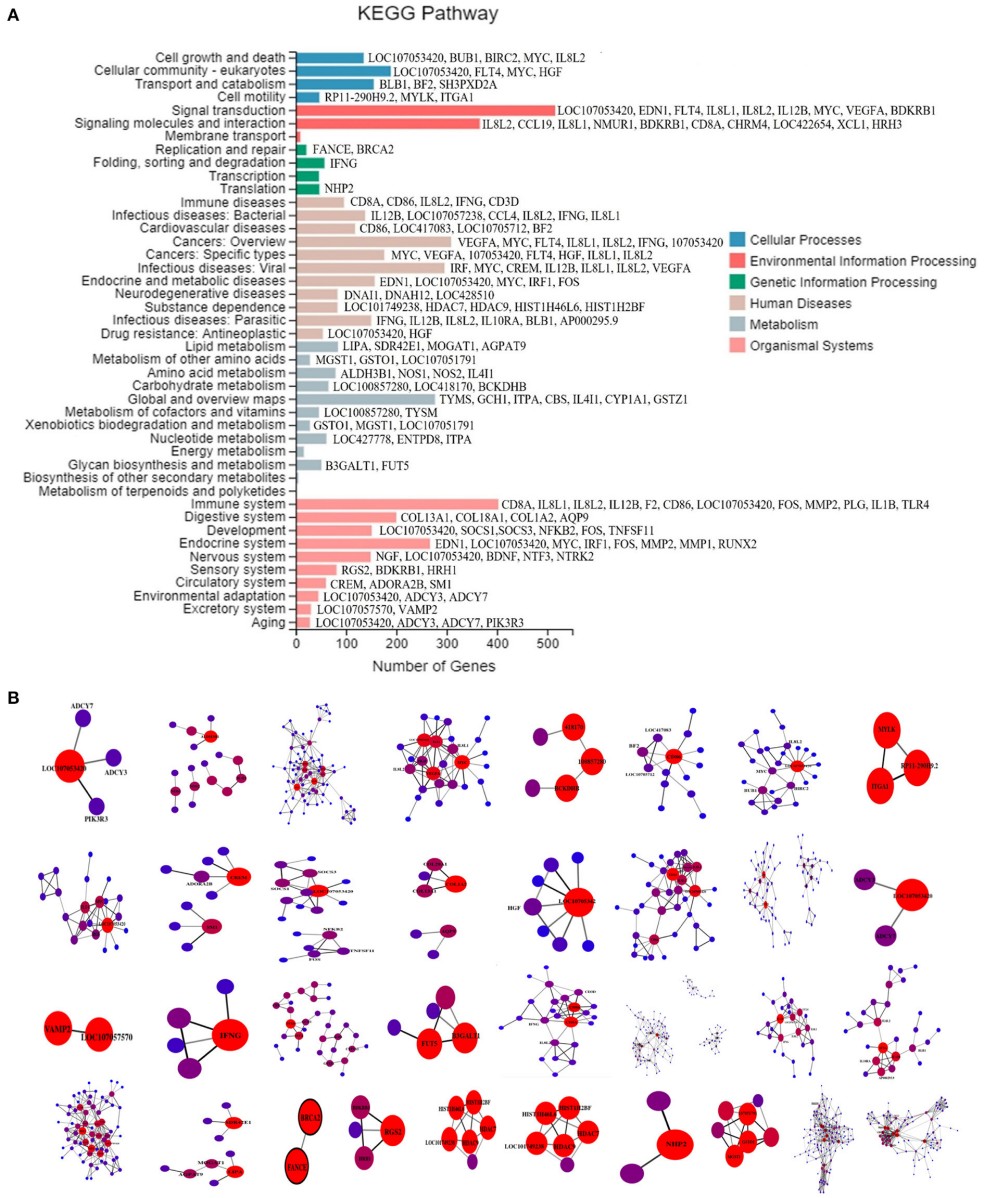
**(A)** KEGG classification map of DEGs. KEGG categorization of the DEGs in the transcriptome of chicken lung induced by co-infection. The X-axis is the number of genes annotated to a KEGG Pathway classification, and the Y-axis is the KEGG Pathway classification. The gene symbols behind the histogram are the target genes screened by PPI. **(B)** PPI maps of DEGs in co-infection group. The Gene marked with Gene Symbol is the target Gene whose interaction relationship is >300. The brighter the color is, the greater the node correlation and the stronger the interaction relationship is.

In this study, multi-omics analyses were performed on the co-infection model as explained previously (23). According to the results of RNA-seq, 3,115 DEGs were found between the co-infection and the control groups, including 1,456 genes upregulated and 1,659 genes downregulated. These DEGs were widely distributed in 44 sub-categories in six major categories in the KEGG pathway database, as shown in Figure 1A. The PPI results in each KEGG classification were submitted in Figure 1B.

### Reverse screening of NCMC in TCMSP database

**Figure 2:**
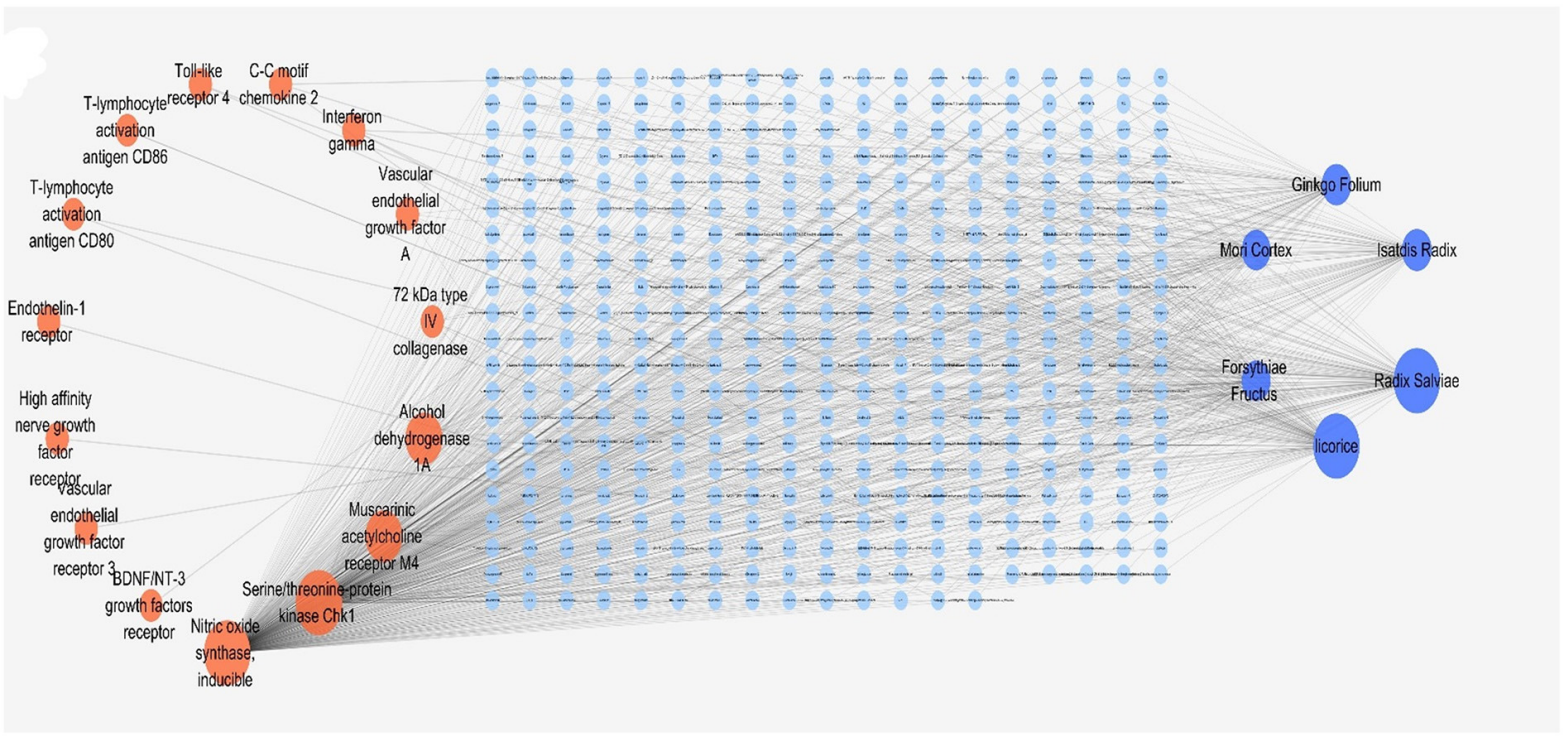
The predicted “target-component-TCM” network. The orange dots were the effective targets, the dark blue dots were the six Chinese medicines obtained by reverse screening, and the light blue dots were the components of Chinese medicines.

We first collected the entire data of the TCMSP Chinese Medicine Database including 499 TCMs and their constituent information (13,889 TCM molecules) and corresponding drug targets (702), and organized them into a TCM network containing 113,265 relationships which was submitted in Supplementary material 1. Fifty-seven target genes screened by PPI were substituted into the Drug bank database (Table 1) and 25 effective targets were obtained. These 25 targets were brought into the TCMSP total network for the reverse screening of Chinese medicine, and the results showed that 15 targets were matched with the TCMSP database (Reverse network as shown in Supplementary material 4). Afterward, we sorted the correlation degree of Chinese medicine nodes and selected six high-ranked Chinese medicines according to the classification of Chinese medicines. The six herbs were *Isatdis Radix, Forsythia Fructus, Ginkgo Folium, Mori Cortex*, Licorice, and *Radix Salviae*, and the final constructed “target-component-TCM” network of TCM compound was shown in Figure 2.

**Table 1:** Targets associated with mixed infection obtained.

**Gene ID**	**Gene symbol**	**DrugBank name**
107053420	GTPase HRas-like	GTPase KRas
396495	IL8L2	High affinity interleukin-8 receptor A
420332	MYC	Proto-oncogene serine/threonine-protein kinase Pim-1
395872	IL8L1	High affinity interleukin-8 receptor A
404671	IL12B	Interleukin-12 subunit beta
395742	FLT4	Vascular endothelial growth factor receptor 3
395909	VEGFA	Vascular endothelial growth factor A
772372	BDKRB1	B2 bradykinin receptor
396054	IFNG	Interferon gamma
420854	EDN1	Endothelin-1 receptor
100857280	ADH1L	Alcohol dehydrogenase 1A
421060	TYMS	Thymidylate synthase
427944	CD86	T-lymphocyte activation antigen CD86
396512	FOS	Proto-oncogene protein c-fos
395941	HGF	Serine protease hepsin
693256	BLB1	Major histocompatibility complex class I
425389	BF2	Major histocompatibility complex class I
418178	MGST1	Microsomal glutathione S-transferase 1
423881	GSTO1	Glutathione transferase omega-1
107051791	MGST2	Microsomal glutathione S-transferase 2
417039	IL4I1	D-amino-acid oxidase
424390	ITPA	NTPase P4
403158	CD8A	T-lymphocyte activation antigen CD80
386583	MMP2	72 kDa type IV collagenase
396384	IRF1	NADH dehydrogenase (ubiquinone) 1 alpha
396445	MYLK	Myosin light chain 6B
423195	CHRM4	Muscarinic acetylcholine receptor M4
428147	HRH3	Histamine H3 receptor
415806	SDR42E1	Oxidoreductase
423789	LIPA	Lipase estA
374209	RP11	Insulin-like growth factor-binding protein 7
423876	SH3PXD2A	NADPH oxidase organizer 1
424811	MOGAT1	Serine acetyltransferase
428813	ALDH3B1	Aldehyde dehydrogenase 3B1
427721	NOS1	Nitric oxide synthase, inducible
395807	NOS2	Nitric oxide synthase oxygenase
395375	BCKDHB	3-methyl-2-oxobutanoate hydroxymethyltransferase
396146	GCH1	GTP cyclohydrolase I
417241	TLR4	Toll-like receptor 4
396243	COLIA2	Short tail fiber protein
396466	NGF	Beta-nerve growth factor
428099	NTF3	High affinity nerve growth factor receptor
396157	NTRK2	BDNF/NT-3 growth factors receptor
419891	FANCE	Serine/threonine-protein kinase Chk1
416218	NHP2	NHP2-like protein 1
395663	AP000295.9	Ribosomal protein S6 kinase alpha-1
419771	IL10RA	C-C motif chemokine 2
417083	-	Interleukin-1 receptor-associated kinase-4
101749238	-	Major histocompatibility complex class I
769973	HIST1H2BF	Histone deacetylase
415732	ADCY7	NADH dehydrogenase 1 alpha subcomplex subunit 13
422013	ADCY3	Mitogen-activated protein kinase 13
424827	VAMP2	Vesicle-associated membrane protein 2
429096	PIK3R3	Phosphatidylinositol 3-kinase regulatory
416630	SOCS1	Proto-oncogene tyrosine-protein kinase MER
386574	NFKB2	Nuclear factor NF-kappa-B p105 subunit
428067	TNFSF11	Tumor necrosis factor ligand superfamily member 11

### Component compounds of NCMC aqueous extraction

**Figure 3:**
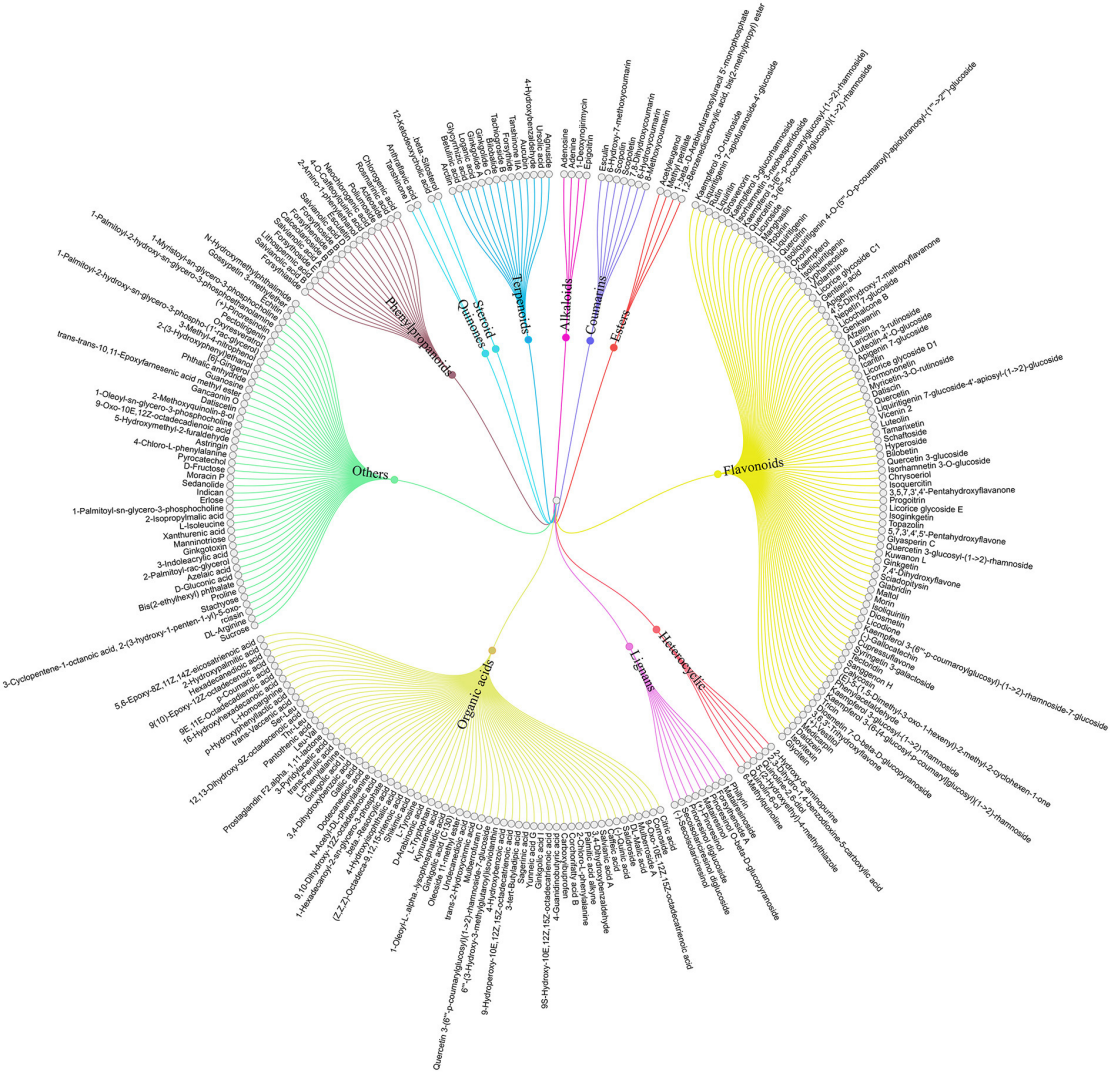
Tree diagram of NCMC aqueous extraction classification. Total 260 compounds belong to 12 categories represented by different colors.

To clarify the effective ingredients of NCMC, a non-targeted metabolomics method was used to study the aqueous extraction. The results showed that 260 compounds (including positive and negative modes) were detected on the receiver side using UPLC-QTOF-MS/MS. Among them, 85 compounds belong to flavonoids, 64 compounds belong to organic acids, and 17 compounds belong to phenylpropanoids, and so on, as shown in Figure 3. The complete composition data of NCMC is provided in Supplementary material 5.

### Pharmacodynamics' evaluation of NCMC

**Figure 4:**
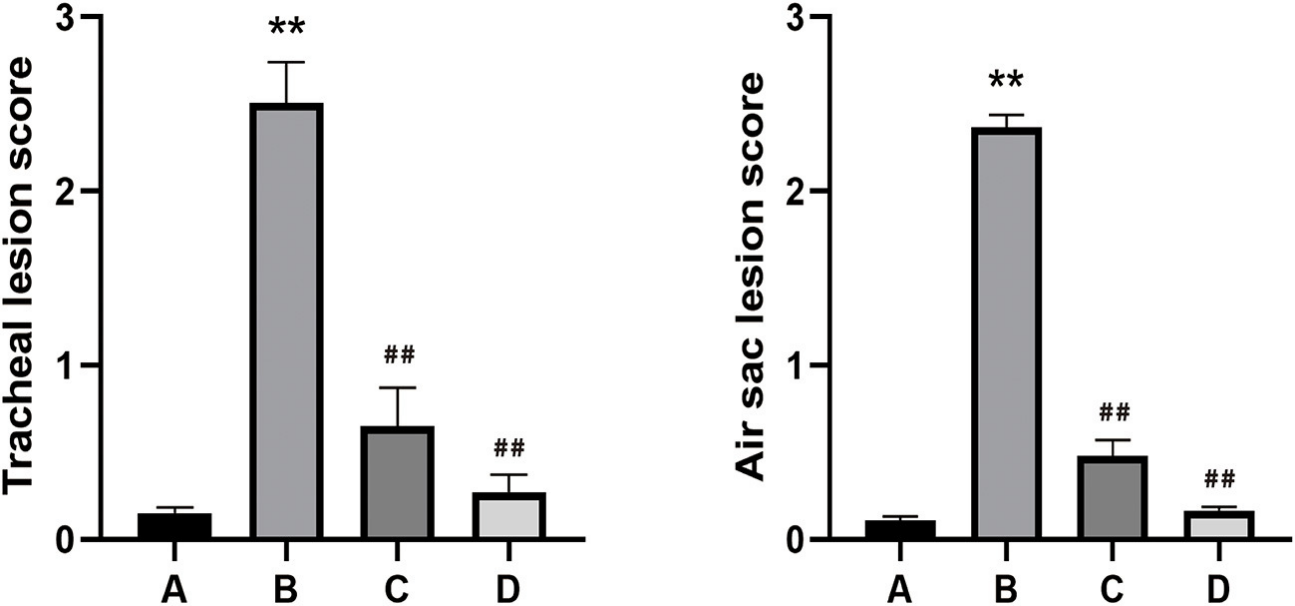
Gross and tracheal lesion evaluation of all groups, **(A)** Control group. **(B)** Co-infection group. **(C)** Co-infection + NCMC administration group. **(D)** NCMC control group. Bar graphs represent mean results ± SD. Statistical significance were represented as ^**^P < 0.05 vs. control group, ^*##*^*P* < 0.05 vs. model group. Error bars represent the standard error of the mean.

**Figure 5:**
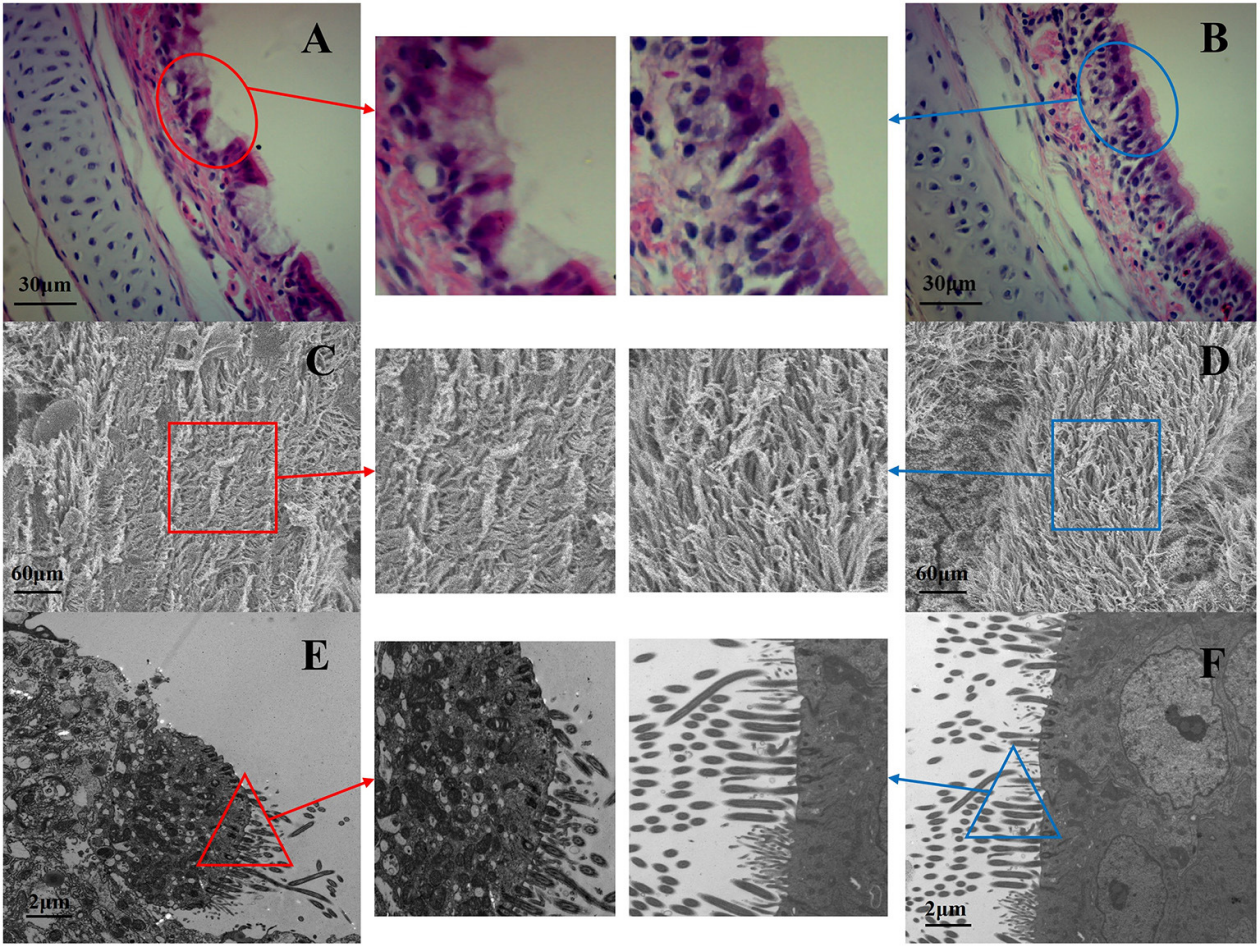
Pathological and ultrastructural changes of NCMC treatment on chicken trachea, (**A,C,E** from the co-infection group; **B,D,F** from the NCMC treatment group). Paraffin sections of tracheal tissues from co-infection group and co-infection + NCMC group were stained with hematoxylin-eosin (100×). **(A)** The red circle shows the cilia are exfoliated and there is a proliferation of goblet cells. **(B)** The blue circle indicates that the cilia are well arranged and only a small amount of inflammatory cell infiltration. SEM of **(C,D)** at a magnification of 2,500×, and TEM of **(E,F)** at a magnification of 8,000×. **(D)** The cilia in blue square significantly more abundant and intact after treatment of co-infection **(C)**. **(E)** The cilia were ruptured, matrix electron density decreased in red triangle of co-infection group. **(F)** The blue triangle shows some cilia were shed, but the symptoms of the treatment group were significantly improved.

The chickens in co-infection group compared with the other three groups had severe air sac lesions with diffuse yellowish thickening with caseous exudates, and the control group had no significant changes. There was no significant difference between A and C for the gross air sac lesion score. The same trend was observed for the tracheal lesion scores in the four groups (Figure 4). Pathological and ultrastructural changes (Figure 5) were performed to better understand the effects of NCMC treatment on the chicken trachea. In the co-infection group (Figure 5A), the cilia are exfoliated and there is a proliferation of goblet cells in the upper mucosa. While the cilia are well arranged and there is still a small amount of inflammatory cells infiltrations after NCMC treatment (Figure 5B). Scanning electron microscopy (SEM) showed that the cilia were significantly more abundant and intact after treatment of co-infection (Figures 5C,D). TEM examination showed that cilia were ruptured, matrix electron density decreased, and the formation of umbrella-like structure to the extracellular in the co-infection group (Figure 5E). Although some cilia were shed, the symptoms of the treatment group were significantly improved compared with the co-infection group (Figure 5F).

## Discussion

In this study, we conducted multi-target screening for co-infection of MG and *E. coli* based on omics research and investigated the polypharmacology mechanism of NCMC with multiple components. The whole research was based on the establishment of a co-infection model and the subsequent target screening. According to the previous transcriptome studies on co-infection (23), we obtained a total of 3,115 DEGs and the corresponding KEGG classification. As the previous study showed that oxidative stress transcriptomics and drug discovery approaches could identify and target neurotoxic innate immune populations and lead to the development of selective neuroprotective strategies (37, 38). However, we hope to screen the representative DEGs from the overall level of *in vivo* studies. Hence, we have carried out the DEGs from 33 sub-categories of KEGG have carried out PPI analysis, respectively, as explained in previous studies (39–41). The screened target genes cannot be applied directly but are matched to the corresponding target according to the Information in the DrugBank database.

Hopkins proposed the concept of network pharmacology in 2007, which aims to design a new generation of drugs by incorporating biological networks rather than single target (42). Network pharmacology-based strategy predicted liver injury targets of matrine against COVID-19 and further confirmed Matrine can maintain liver function homeostasis, regulate immunity and antivirus through direct target action and signal pathway regulation in *vivo* (43). For complex diseases, the method of applying network pharmacology was also used to reveal the mechanism of action of TCM (44, 45). We collected the whole data of TCMSP for the construction of the total network “TCM-component-target”, and then the targets were substituted into the total network for the reverse screening of TCMs. This method of reverse operation is similar to existing relevant studies (46, 47). Our study combined the usual methods of network pharmacology in the past, starting from the complex diseases in clinical practice, and re-screening TCM compounds through the study of natural products. However, this is the first attempt to apply the reverse screening method directly to the TCM network.

After obtaining the order of Chinese herbs, we then selected different categories based on the compatibility theory of Chinese medicines. According to the degree of node correlation, the order was as follows: *Isatdis Radix* (4th, yang tonic) (48), *Forsythia Fructus* (11th, Antipyretic and antidote) (49), *Ginkgo Folium* (5th, Antitussive and antiasthmatic drugs) (50), *Mori Cortex* (3rd, Antitussive and antiasthmatic drugs) (51), Licorice (1st, reinforcing drugs) (52), and *Radix Salviae* (2nd, Drugs with the efficacy of modifying rheological properties of blood) (53). Aqueous extract of Chinese medicinal compound is the most common method to study compound components (54, 55). Hence, we explored the optimization design studies (56, 57). In preliminary experiments, a multi-nonlinear regression analysis was used for searching the optimal combination based on the minimal inhibitory concentration of MG and *E. coli in vitro*, air sac and tracheal lesion scores *in vivo*. Then, the different proportion combinations were further optimized through uniform design. Finally, we obtained the optimal combination of NCMC aqueous extract for subsequent targeted therapy experiments. In the treatment experiment of NCMC aqueous extract for co-infection, multiple pathological methods were employed to observe whether a certain curative effect was achieved. Pathological and ultrastructural results showed that the symptoms of co-infection were significantly relieved after treatment, which indicated that NCMC played a significant therapeutic role.

In summary, the target network for the co-infection was dug through the joint analysis of transcriptomics and metabolomics, and the mapping between the target network and the total TCM network was used to reverse screen TCMs. We started from the network and carried out targeted verification from multiple dimensions to preliminarily reveal the polypharmacological effects of NCMC. These data suggest that we may provide a new method to validate target combinations of natural products, which perhaps to optimize their multiple structure-activity relationships to obtain drug-like natural product derivatives. The research on the product of new TCM formula provides direction and guidance for the development of drugs for respiratory diseases in veterinary clinic.

## Data availability statement

The original contributions presented in the study are included in the article/Supplementary material, further inquiries can be directed to the corresponding authors.

## Ethics statement

The animal study was reviewed and approved by Laboratory Animal Ethics Committee of Northeast Agricultural University.

## Author contributions

ZWu and JL designed the study. JB, YW, SW, DN, ZWu, YZ, and RL performed and collected data from experiment and analyzed data. ZWu and MI wrote the manuscript. All authors read and approved the final manuscript.

## Funding

This work was supported by the National Natural Science Foundation of China (32273062) and Special Funding for Postdoctoral of Heilongjiang Province (LBH-TZ2119).

## Conflict of interest

The authors declare that the research was conducted in the absence of any commercial or financial relationships that could be construed as a potential conflict of interest.

## Publisher's note

All claims expressed in this article are solely those of the authors and do not necessarily represent those of their affiliated organizations, or those of the publisher, the editors and the reviewers. Any product that may be evaluated in this article, or claim that may be made by its manufacturer, is not guaranteed or endorsed by the publisher.
