# Supplementary material for: Polypharmacology-based approach for screening TCM against coinfection of Mycoplasma gallisepticum and Escherichia coli
Source: Front Vet Sci. 2022 Sep 26;9:972245. doi: 10.3389/fvets.2022.972245 (PMC9549337; doi:10.3389/fvets.2022.972245)
Supplement: Supplementary file 1 [file Data_Sheet_1.ZIP › Supplementary materials/Supplementary materials3.docx]

**The design optimization of Chinese medicinal compound for the treatment of co-infection of *Mycoplasma gallisepticum* and *Escherichia Coli***

**Zhiyong Wu^1^, Qianqian Fan^1^, Jiaxin Bao^1^, Rui Li^1^, Muhammad Ishfaq^1,*^, Jichang Li^1,^****^2,*^**

**^1^**College of Veterinary Medicine, Northeast Agricultural University, 600 Changjiang Road, Xiangfang District, Harbin 150030, P. R. China. **^2^**Heilongjiang Key Laboratory for Animal Disease Control and Pharmaceutical Development, 600 Changjiang Road, Xiangfang District, Harbin 150030, P. R. China.

^*^Corresponding authors at: College of Veterinary Medicine, Northeast Agricultural University, 600 Changjiang Road, Xiangfang District, Harbin 150030, P. R. China.

Jichang Li. Telephone: +86 451 5519 0674. Fax: +86 451 5519 1200. E-mail: [lijichang@neau.edu.cn](mailto:lijichang@neau.edu.cn)

**Running Head:** Design optimization of Chinese medicinal compound

**Abstract**

**Background:** A new Chinese medicinal compound including Isatdis Radix, Forsythiae Fructus, Ginkgo Folium, Mori Cortex, Licorice and Radix Salviae was pre-screened through the method of network pharmacology for the treatment of co-infection of *Mycoplasma gallisepticum* and *Escherichia Coli*. While, the optimal combination of Chinese medicine is still an obstacle to the development of the Chinese medicinal compound.

**Results:** A multi-indicators evaluation method was used in this study to establish a comprehensive assessment system based on the minimal inhibitory concentration of *Mycoplasma gallisepticum* and *Escherichia Coli* *in vitro*, air sac and tracheal lesion scores *in vivo*. We used the min-max normalization method to normalize the scores of the four metrics and gave them the same proportion of weights to calculate the total score. Then, according to the method of uniform test, the six-factor ten-level experiment (*U*^*^_10_(10^6^) was designed, with the total score as the index. The multi-nonlinear regression equation was established by data processing system according to the comprehensive index. The results showed that the ratio of Isatdis Radix, Forsythiae Fructus, Ginkgo Folium, Mori Cortex, Licorice and Radix Salviae were 14:7:11:12:5:3, which showed effective treatment.

**Conclusions:** It turned out that uniform design can be effectively used to solve dose optimization for combined use of multiple compounds. In addition, this study also proposes a new method to comprehensively evaluate medicinal compounds for respiratory diseases such as *Mycoplasma gallisepticum* and *Escherichia Coli* infection.

**Key words:** Chinese medicinal compound; Uniform design; Multi-index; *Mycoplasma gallisepticum*; *Escherichia Coli*

**Background**

Studies demonstrated that respiratory diseases cause major economic losses in poultry production worldwide [[1](#_ENREF_1)]. It is worthy to mention that when multiple infections occur, the mortality rate of infected chickens increased dramatically [[2](#_ENREF_2), [3](#_ENREF_3)]. In recent years, the threat of co-infection is growing with the expansion of intensive farming. However, there are few reports on the study of drug research for the treatment of co-infection.

Traditional Chinese medicine (TCM) is increasingly used around the world due to its low-toxicity and better efficacy, but the evidence on its effectiveness is a matter of debate [[4](#_ENREF_4)]. However, with the rapid development of high-throughput industry and network pharmacology recent years, the network target-based approaches may provide a systematic paradigm for facilitating the development of multicomponent therapeutics and the modernization of TCM [[5](#_ENREF_5), [6](#_ENREF_6)]. We have selected six TCMs according to this method, while the optimal combination of Chinese medicine is still an obstacle to the development of Chinese medicinal compound (CMC). In this study, we focus on the strategy for optimizing the combination of active components of CMC based on our laboratory's model research [[7](#_ENREF_7)] .

A *Uniform Design* (*UD*) was proposed by Prof. Fang and mathematician Wang Yuan in 1978, and it is an application of the "pseudo-Monte Carlo method" [[8](#_ENREF_8)]. Cai found that the experimental optimization and analysis methods (*UD*) can be effectively used to solve both compatibility and dose optimization for combined use of multiple compounds [[9](#_ENREF_9)].The steps for *UD* are mainly including the choice of a suitable *UD* table related to the number of factors and levels, and the establishment of a regression [[10](#_ENREF_10)]. Meanwhile, four universally accepted indexes were used: air sac and tracheal lesion scores, the minimal inhibitory concentration (MIC) of *MG* and *E.coli* [[11](#_ENREF_11), [12](#_ENREF_12)]. These four indexes were widely used to assess the infection. We aggregated these four metrics into a total score as a final index of the effectiveness of the CMC. In this trial, we sought design points which were uniformly scattered on the domain, furthermore we did a multi-nonlinear regression analysis to search the optimal combination.

**Materials and methods**

**Animal, Mycoplasma strains and Bacteria**

120 commercial Leghorn chickens were obtained from Chia Chau Chicken Farm (Harbin, Heilongjiang, China), which did not undergo vaccination and raised to day 7. The *MG* strain R_low_ was obtained from Harbin Institute of Veterinary Medicine, Chinese Academy of Agricultural Science. The mycoplasmas were cultured at 37 ℃ as described previously [[13](#_ENREF_13)]. *MG* was used to challenge chickens at the density of 1×10^9^ CCU/ml (color change unit per milliliter) in the culture medium. *E.coli* O78, was isolated from chickens infected with colibacillosis in our laboratory and cultured in Mueller-Hinton Broth (Beijing Aoboxing BIO-TECH CO., LTD). The concentration of *E.coli* was adjusted to 10^9^ CFU/ml before infection.

**Chicken infection and groupings**

A total of 120 White Leghorn chickens were purchased from Chia Chau Chicken Farm (Harbin, Heilongjiang, China). The infectious dose was diluted into 0.2 ml *MG* medium in left caudal thoracic air sac at the 7^th^ day, and injected intraperitoneally with 0.1 ml of *E.coli* at day 10 in chickens. From the 13^th^ day, 120 chickens were randomly divided into 10 groups, of which 10 groups were treated with different CMC (Group A-J). The chickens of treatment groups were given as the dose of 450 mg/kg continuously by intragastric administration for a week [[14](#_ENREF_14)].

***UD* design**

The six Chinese herbs were purchased from Runhe Chinese medicine processing plant Ltd. (Harbin, China) and were selected as the six factors and each factor set 10 levels for *UD*. The minimum dose range for each TCM is 0 and the highest value refers to the use and dosage as mentioned in Chinese Pharmacopoeia by State Pharmacopoeia Committee (Edition, 2015). As shown in **Table.1**, the experiment was arranged following the uniform table *U*^*^_10_(10^6^). The method of water extraction is described previously [[15](#_ENREF_15)].

**Standardization of evaluation index and calculating comprehensive index**

The min-max normalization method was adopted to eliminate the effects of different dimension [[16](#_ENREF_16)]. Four indices were used to calculate the total score, including the MIC of *MG* and *E.coli* in vitro, air sac and tracheal lesion scores in vivo, and these four were standardized using the formula (1). The composite score was calculated following formula (2).

(1). $R_{i}=\frac{X_{max}-X_{i}}{X_{max}-X_{min}}\times100$

i represents sample serial number; *R_i_* is non-dimensionalized value after conversion by min-max normalization method; x_i_ is the i^th^ measured value, x_max_ is the maximum of all measured values, x_min_ is the minimum of all measured values.

(2). $D=\sum0.25R_{i}$

D represents the comprehensive evaluation index; *R_i_* is nondimensionalized value after conversion by min-max normalization method.

Evaluation rules for respiratory diseases assessment

The severity of the gross air sac lesions was scored on a scale of 0 - 3 as described previously [[17](#_ENREF_17)]. The tracheal lesion score was determined from the same three discontinuities of the trachea. The tracheal infection score was also calculated on the basis of 0 to 3 scoring system [[18](#_ENREF_18)]. The MIC of *MG* and *E.coli* in vitro were divided into 10 serial dilutions ranged from 640 μg/mL to 1.25 μg/mL. We define the results based on the number of plates that detect MIC and calculate them in formula (1).

**MIC determination**

MIC was determined using a microdilution technique with *MG* titers of 2 × 10^5^ CCU/mL. A 0.1 mL aliquot of that media was added to a 96-well plate and another 0.1 mL CMC (1280 μg/mL) was added to the first plate. A series of concentrations of CMC were diluted by doubling (Range from 640 μg/mL to 1.25 μg/mL) and the last 0.1 ml discarded. Another 0.1 mL aliquot of prepared *MG* was supplemented to each well, achieving the final titers of 1 × 10^5^ CCU/mL. The above method is the same to the test of *E.coli*. Tests were conducted in triplicate and the method of determination was used as described previously [[12](#_ENREF_12), [19](#_ENREF_19)].

**Statistical analysis**

Data are presented as mean results ± standard deviation (SD). Multi-nonlinear regression equation was made by the method of secondary polynomial gradual regression in DPS software [[20](#_ENREF_20)].

**Results**

**Measured data of assessment indicators**

The results of the four indicators are summarized in Table.2. It is difficult to carry out comprehensive analysis and evaluation when applying multi-indicators assessment, because of the different dimensions of indicators and the value of different indicators [[21](#_ENREF_21)]. Therefore, the data of each evaluation indicator needed to be dimensionless, as shown in **Table.2**.

**The optimal combination of principal constituent analysis by *UD***

It is necessary to have a uniform experimental design to filter out the best combination, further to combine the comprehensive effect with the multi-indicator analysis. In this study, Isatdis Radix (X_1_), Forsythiae Fructus (X_2_), Ginkgo Folium (X_3_), Mori Cortex (X_4_), Licorice (X_5_) and Radix Salviae (X_6_) were chose to be the principal constituents by *UD*. The *U*^*^_10_(10^6^) *UD* table was used to design an experiment of 10 combinations. The composite score as a dependent variable, represented by Y. The multivariate quadratic equation was established by DPS: Y = -223.2+57.8×X_2_+0.5×X_4_+46.4×X_5_+0.1×X_1_^2^-2.9×X_2_^2^-2.0×X_5_^2^+0.002×X_2_×X_3_-4.1×X_2_×X_5_, R^2^ = 0.999998, F value = 62499.9063, Regression significance test (p = 0.0031). The results showed that the ratio were 14:7:11:12:5:3, which has the best treatment effect. All analysis results of DPS are recorded in supplementary material.

**Discussion**

The optimization design of multi-component drug based on the active ingredients of TCM is an effective way to inherit the advantages of TCM formulation and develop modern TCM [[22](#_ENREF_22)]. As the complexity of components in TCM formulation, the application of statistical experimental design methods and mathematical models may greatly help to effectively search for effective drug combinations [[23](#_ENREF_23)]. Hence, the method of *UD* and multi-indexes evaluation were used in the traditional Chinese medicine treatment experiment in this study.

In the *UD*, the minimum dose was 0, which indicated that the drug was blank, and the highest value was referred to the use and dosage as mentioned in Chinese Pharmacopoeia by State Pharmacopoeia Committee (Edition, 2015). We divided the available doses of six herbs into ten levels to match the uniform test table *U*^*^_10_(10^6^), which referred to the previous studies [[8](#_ENREF_8)]. The *UD* table does not have repeated numbers in each column, and the ordered number pairs formed by any two columns of peer numbers are different, and each number pair appears only once.

Our results showed that the multivariate quadratic equation as follows: Y = -223.2+57.8×X2+0.5×X4+46.4×X5+0.1×X12-2.9×X22-2.0×X52+0.002×X2×X3-4.1×X2×X5, R2 = 0.999998, F value = 62499.9063, Regression significance test (p = 0.0031). The equation can better fit the effect of each CMC on the total score, which suggests that Isatdis Radix, Mori Cortex and Licorice have a positive correlation. From the results of the regression equation, it can be seen that the influence of Isatdis Radix on the total score is the greatest.

**Conclusions**

In summary, this study used a *UD* method to optimize the different combinations of TCM and carried out multiple secondary regression according to the comprehensive index, which might provide some evidences for further development of new modern Chinese drug and comprehensive evaluation of respiratory diseases in poultry.

**Abbreviations**

Chinese medicinal compound (CMC); minimal inhibit concentration (MIC); Mycoplasma gallisepticum (MG); Escherichia Coli (E.coli); data processing system (DPS); uniform design (UD); traditional Chinese medicine (TCM); color change unit (CCU); Colony-Forming Units (CFU).

**Acknowledgements**

The authors would like to thank all participants in this project for their technical assistance, especially Prof. Ming Ge (Northeast Agricultural University, China).

**Authors’ contributions**

ZW and JL designed the study. QF, JB and RL performed and collected data from experiment and analyzed data. ZW, IM wrote the manuscript. All authors read and approved the final manuscript.

**Funding**

This work was supported by the National Natural Science Foundation of China (31973005).

**Availability of data and materials**

The data sets used and analysed during the current study are available from the corresponding author on reasonable request.

**Ethics approval and consent to participate**

The present study was conducted under the approval of Laboratory Animal Ethics Committee of Northeast Agricultural University (Heilongjiang province, China) in accordance with Laboratory animal-Guideline for ethical review of animal welfare (GB/T 35892-2018, National Standards of the People's Republic of China).

**Consent for publication**

Not applicable.

**Competing interests**

The authors declare no conflicts of interest and no competing financial interests.

**References**

1. Sid, H., K. Benachour, and S. Rautenschlein, Co-infection with Multiple Respiratory Pathogens Contributes to Increased Mortality Rates in Algerian Poultry Flocks. Avian Dis, 2015. **59**(3): p. 440-6.

2. Stipkovits, L., et al., Pathologic lesions caused by coinfection of Mycoplasma gallisepticum and H3N8 low pathogenic avian influenza virus in chickens. Vet Pathol, 2012. **49**(2): p. 273-83.

3. Sid, H., et al., Mycoplasma gallisepticum modifies the pathogenesis of influenza A virus in the avian tracheal epithelium. Int J Med Microbiol, 2016. **306**(3): p. 174-86.

4. Shang, A., et al., Placebo-controlled trials of Chinese herbal medicine and conventional medicine comparative study. Int J Epidemiol, 2007. **36**(5): p. 1086-92.

5. Hopkins, A.L., Network pharmacology. Nat Biotechnol, 2007. **25**(10): p. 1110-1.

6. Li, S., B. Zhang, and N. Zhang, Network target for screening synergistic drug combinations with application to traditional Chinese medicine. BMC Syst Biol, 2011. **5 Suppl 1**: p. S10.

7. Wu, Z., et al., Co-infection of Mycoplasma gallisepticum and Escherichia coli Triggers Inflammatory Injury Involving the IL-17 Signaling Pathway. 2019. **10**(2615).

8. Fang, K.T., et al., Uniform Design: Theory and Application. Technometrics, 2000. **42**(3): p. 237-248.

9. Cai, C., et al., Synergistic Effect of Compounds from a Chinese Herb: Compatibility and Dose Optimization of Compounds from N-Butanol Extract of Ipomoea stolonifera. Sci Rep, 2016. **6**: p. 27014.

10. Ning, J.-H., K.-T. Fang, and Y.-D. Zhou, Uniform Design for Experiments with Mixtures. Communications in Statistics, 2011. **40**(10): p. 1734-1742.

11. Leigh, S.A., et al., Effect of infection route and concurrent infectious bronchitis virus vaccination on Mycoplasma gallisepticum disease pathology in an experimental model. Avian Pathol, 2012. **41**(5): p. 497-503.

12. Xiao, X., et al., Ex vivo pharmacokinetic and pharmacodynamic analysis of valnemulin against Mycoplasma gallisepticum S6 in Mycoplasma gallisepticum and Escherichia coli co-infected chickens. Vet J, 2015. **204**(1): p. 54-9.

13. Lu, Z., et al., TLR2 mediates autophagy through ERK signaling pathway in Mycoplasma gallisepticum-infected RAW264.7 cells. Mol Immunol, 2017. **87**: p. 161-170.

14. Wu, Z., et al., Baicalin Attenuates Mycoplasma gallisepticum-Induced Inflammation via Inhibition of the TLR2-NF-kappaB Pathway in Chicken and DF-1 Cells. Infect Drug Resist, 2019. **12**: p. 3911-3923.

15. Peter, K., et al., A novel concept for detoxification: complexation between aconitine and liquiritin in a Chinese herbal formula ('Sini Tang'). J Ethnopharmacol, 2013. **149**(2): p. 562-9.

16. Juan, D.U. and Y. Chen, Ecosystem Health Evaluation of Oasis-Desert Ecotone in Minqin County Based on PSR Model. Research of Soil & Water Conservation, 2016.

17. Ferguson, N.M., V.A. Leiting, and S.H. Klevena, Safety and efficacy of the avirulent Mycoplasma gallisepticum strain K5054 as a live vaccine in poultry. Avian Dis, 2004. **48**(1): p. 91-9.

18. Sprygin, A.V., et al., Biological characterization of Russian Mycoplasma gallisepticum field isolates. Avian Pathol, 2011. **40**(2): p. 213-9.

19. Xiao, X., et al., Mycoplasma gallisepticum and Escherichia coli mixed infection model in broiler chickens for studying valnemulin pharmacokinetics. J Vet Pharmacol Ther, 2014. **37**(1): p. 99-102.

20. Tang, Q.Y. and C.X. Zhang, Data Processing System (DPS) software with experimental design, statistical analysis and data mining developed for use in entomological research. Insect Sci, 2013. **20**(2): p. 254-60.

21. Liang, R., et al., Comprehensive assessment of regional selenium resources in soils based on the analytic hierarchy process: Assessment system construction and case demonstration. Sci Total Environ, 2017. **605-606**: p. 618-625.

22. Li, F., et al., A Strategy for Optimizing the Combination of Active Components Based on Chinese Medicinal Formula Sheng-Mai-San for Myocardial Ischemia. Cell Physiol Biochem, 2018. **45**(4): p. 1455-1471.

23. Zhang, K., et al., Essential oil-mediated glycerosomes increase transdermal paeoniflorin delivery: optimization, characterization, and evaluation in vitro and in vivo. Int J Nanomedicine, 2017. **12**: p. 3521-3532.

**Table 1**. Arrangement of *UD* (*U*^*^_10_(10^6^).

| Group | Isatdis Radix  (X1) | Forsythiae Fructus  (X2) | Ginkgo Folium  (X3) | Mori Cortex  (X4) | Licorice  (X5) | Radix Salviae  (X6) |
| --- | --- | --- | --- | --- | --- | --- |
| A | 0 | 1.5 | 2.4 | 4.8 | 6 | 13.5 |
| B | 1.5 | 4.5 | 6 | 10.8 | 2 | 12 |
| C | 3 | 7.5 | 9.6 | 3.6 | 9 | 10.5 |
| D | 4.5 | 10.5 | 0 | 9.6 | 5 | 9 |
| E | 6 | 13.5 | 3.6 | 2.4 | 1 | 7.5 |
| F | 7.5 | 0 | 7.2 | 8.4 | 8 | 6 |
| G | 9 | 3 | 10.8 | 1.2 | 4 | 4.5 |
| H | 10.5 | 6 | 1.2 | 7.2 | 0 | 3 |
| I | 12 | 9 | 4.8 | 0 | 7 | 1.5 |
| J | 13.5 | 12 | 8.4 | 6 | 3 | 0 |

According to the method of uniform test, the six-factor ten-level experiment (*U*^*^_10_(10^6^) was designed. The minimum dose was 0 and the highest value was referred to Chinese Pharmacopoeia by State Pharmacopoeia Committee (Edition, 2015).

**Table 2**. Results of the four indicators, including the MIC of *MG* and *E.coli*, air sac and tracheal lesion scores.

| Group | MIC of MG | MIC of E.coli | air sac lesion | tracheal lesion | Total score^a^ |
| --- | --- | --- | --- | --- | --- |
|  | μg/mL(R_i_) | μg/mL(R_i_) | Mean±SD(R_i_) | Mean±SD(R_i_) |  |
| A | 40(50) | 640(10) | 2.40±0.52(20.0) | 1.95±0.90(35.0) | 28.75 |
| B | 40(50) | 640(10) | 1.70±1.06(43.3) | 1.85±0.94(38.3) | 35.42 |
| C | 40(50) | 320(20) | 2.20±1.03(26.7) | 2.15±0.88(28.3) | 31.25 |
| D | 20(60) | 320(30) | 1.80±1.14(40.0) | 1.80±1.01(40.0) | 42.50 |
| E | 80(40) | 160(30) | 2.40±0.97(20.0) | 2.40±0.70(20.0) | 27.50 |
| F | 80(40) | 640(10) | 2.20±1.32(26.7) | 1.85±0.94(38.3) | 28.75 |
| G | 20(60) | 320(30) | 2.30±0.82(23.3) | 1.80±0.89(40.3) | 38.42 |
| H | 20(60) | 320(20) | 2.10±0.88(30.0) | 2.05±0.96(31.7) | 35.42 |
| I | 10(70) | 160(30) | 1.50±1.08(50.0) | 1.15±0.78(61.7) | 52.92 |
| J | 20(60) | 80(40) | 1.40±1.35(53.3) | 1.35±1.00(55.0) | 52.08 |

R_i_ in parentheses is the result of the non-dimensionalization of the corresponding score according to formula (1). Total score^a^ is calculated by R_i_ according to formula (2).
